# An Evaluation of High Dose Antipsychotic Therapy Prescribing Across the General Adult Inpatient Wards and the Psychiatric Intensive Care Unit in Mersey Care NHS Foundation Trust

**DOI:** 10.1192/bjo.2024.579

**Published:** 2024-08-01

**Authors:** Declan Hyland, Roopa Singh, Kerry Dainton

**Affiliations:** Mersey Care NHS Foundation Trust, Liverpool, United Kingdom

## Abstract

**Aims:**

High Dose Antipsychotic Therapy (HDAT) should only be used in exceptional circumstances, as there is little evidence to suggest that higher than recommended doses of antipsychotics are more clinically effective than standard doses, with potential side effects being greater. In practice, there are several clinical scenarios where HDAT may be prescribed and the potential benefits must outweigh the potential risks. NICE guidelines for psychosis and schizophrenia advise that dosages outside the range given in the British National Formulary should be justified and recorded.

This evaluation aimed to determine the prescribing practice involved with HDAT across the 16 general adult inpatient wards and Psychiatric Intensive Care Unit (PICU) in Mersey Care NHS Foundation Trust.

**Methods:**

A list of all inpatients on the 16 general adult inpatient wards and the PICU in the Trust between 17^th^ and 20^th^ of July 2023 was obtained. Each patient's electronic prescription record was scrutinised to determine whether the patient was prescribed HDAT. For each HDAT patient, the patient's electronic psychiatric record was reviewed to determine whether the decision to be prescribed HDAT was authorised by a Consultant, and whether there was evidence of this decision being discussed at a multi-disciplinary team (MDT) meeting and/or patient ward review. The authors also reviewed whether the clinical rationale for the patient to be prescribed HDAT was documented in the patient's clinical record and whether there was documentation of whether the patient had capacity to consent to being prescribed HDAT.

**Results:**

Of the 29 HDAT patients identified, the decision to prescribe HDAT was authorised by a Consultant in 22 (76%) patients. In 14 (48%) patients, the decision to prescribe HDAT was discussed in an MDT meeting and/or patient ward review. The clinical rationale for being prescribed HDAT was documented in 15 (52%) patients. There was evidence of documentation of whether the patient had capacity to consent to being prescribed HDAT in only 8 (28%) patients.

**Conclusion:**

The decision to prescribe HDAT should always be senior-led and involve MDT discussion, to enable input from medical, nursing and pharmacy staff. Current practice across the Trust's general adult inpatient wards and the PICU indicates that work needs to be done to ensure that, in every case of an inpatient needing to be subject to HDAT, the clinical rationale for this is documented. Capacity to consent to being prescribed HDAT must be documented for each HDAT patient as a matter of good clinical practice.

